# Naringenin targets ERK2 and suppresses UVB‐induced photoaging

**DOI:** 10.1111/jcmm.12780

**Published:** 2016-02-10

**Authors:** Sung Keun Jung, Su Jeong Ha, Chang Hwa Jung, Yun Tai Kim, Hoo‐Keun Lee, Myoung Ok Kim, Mee‐Hyun Lee, Madhusoodanan Mottamal, Ann M. Bode, Ki Won Lee, Zigang Dong

**Affiliations:** ^1^ The Hormel InstituteUniversity of MinnesotaMinnesotaMNUSA; ^2^ Division of Functional Food ResearchKorea Food Research InstituteSeongnamKorea; ^3^ College of PharmacyGachon UniversityIncheonKorea; ^4^ China‐US (Henan) Hormel Cancer InstituteZhengzhouHenanChina; ^5^ WCU Biomodulation MajorDepartment of Agricultural Biotechnology and Center for Food and BioconvergenceSeoul National UniversitySeoulKorea; ^6^ Advanced Institutes of Convergence TechnologySeoul National UniversitySuwonKorea; ^7^ Research Institute of Bio Food IndustryInstitute of Green Bio Science and TechnologySeoul National UniversityPyeongchangKorea

**Keywords:** naringenin, photoaging, extracellular signal‐regulated kinase 2, Fos‐related antigen 1, matrix metalloproteinase‐1

## Abstract

A number of natural phytochemicals have anti‐photoaging properties that appear to be mediated through the inhibition of matrix metalloproteinase‐1 (MMP‐1) expression, but their direct target molecule(s) and mechanism(s) remain unclear. We investigated the effect of naringenin, a major flavonoid found in citrus, on UVB‐induced MMP‐1 expression and identified its direct target. The HaCaT human skin keratinocyte cell line and 3‐dimensional (3‐D) human skin equivalent cultures were treated or not treated with naringenin for 1 hr before exposure to UVB. The mechanism and target(s) of naringenin were analysed by kinase assay and multiplex molecular assays. Dorsal skins of hairless mice were exposed to UVB 3 times per week, with a dose of irradiation that was increased weekly by 1 minimal erythema dose (MED; 45 mJ/cm^2^) to 4 MED over 15 weeks. Wrinkle formation, water loss and water content were then assessed. Naringenin suppressed UVB‐induced MMP‐1 expression and AP‐1 activity, and strongly suppressed UVB‐induced phosphorylation of Fos‐related antigen (FRA)‐1 at Ser265. Importantly, UVB irradiation‐induced FRA1 protein stability was reduced by treatment with naringenin, as well as with a mitogen‐activated protein kinase (MEK) inhibitor. Naringenin significantly suppressed UVB‐induced extracellular signal‐regulated kinase 2 (ERK2) activity and subsequently attenuated UVB‐induced phosphorylation of p90^RSK^ by competitively binding with ATP. Constitutively active MEK (CA‐MEK) increased FRA1 phosphorylation and expression and also induced MMP‐1 expression, whereas dominant‐negative ERK2 (DN‐ERK2) had opposite effects. U0126, a MEK inhibitor, also decreased FRA1 phosphorylation and expression as well as MMP‐1 expression. The photoaging data obtained from mice clearly demonstrated that naringenin significantly inhibited UVB‐induced wrinkle formation, trans‐epidermal water loss and MMP‐13 expression. Naringenin exerts potent anti‐photoaging effects by suppressing ERK2 activity and decreasing FRA1 stability, followed by down‐regulation of AP‐1 transactivation and MMP‐1 expression.

## Introduction

Epidemiological evidence and molecular studies suggest that exposure of the skin to ultraviolet (UV) light (*i.e*. sunlight) can induce photoaging 1, 2, 3, 4, 5. Photoaged skin is biochemically characterized by a dramatic decrease in collagen and increases in matrix metalloproteinase‐1 (MMP‐1), or interstitial collagenase, which is considered the major collagenase involved in photoaging in response to UV irradiation 3. In normal biological processes such as embryonic development, organ morphogenesis, wound healing and angiogenesis, MMP‐1 is precisely controlled by tissue inhibitors of MMPs 3. Chronic exposure of the skin to UV, however, causes abnormal MMP‐1 expression 1. Once activated, MMP‐1 directly binds to collagen and degrades it to gelatin, which is then further decomposed by a gelatinase such as MMP‐2 or ‐9 6. The results of an earlier study have shown that low‐dose UV irradiation can induce MMP‐1 and wrinkle formation, and inhibition of MMP‐1 expression by the down‐regulation of AP‐1 with all‐trans retinoic acid (ATRA) consequently prevents UVB‐induced photoaging 1.

Fos‐related antigen 1 (FRA1), a member of the Fos family (c‐Fos, FosB and FRA2), dimerizes with Jun family proteins (c‐Jun, Jun D, and Jun B) to form the activator protein‐1 (AP‐1) transcription factor complex 7, which regulates cell survival, proliferation, motility, and invasiveness 7, 8, 9. Fos‐related antigen 1 plays an important role in the formation of AP‐1 complexes, which regulate the expression of specific genes such as MMP‐1 8, 10. Protein stability is a major regulatory outcome of FRA1 protein expression, and extracellular signal‐regulated kinase (ERK)1/2 signalling is an important mediator of FRA1 protein stability 7. UVB irradiation increases not only ERK1/2 and AP‐1 activity in JB6 P+ cells 11, but also FRA1 expression and AP‐1 DNA‐binding activity in human HaCaT keratinocytes 2. Extracellular signal‐regulated kinases play a critical role in numerous cellular processes, including proliferation, differentiation, survival and motility 9. In a conditional knockout system, ERK2 was revealed to be involved in memory and learning 12. Extracellular signal‐regulated kinase 1 knockout mice are viable, fertile and of normal size, but exhibit a twofold reduction in mature thymocytes 13. However, a critical role for ERK1/2 in UV‐induced MMP‐1 expression has not been clearly demonstrated because of the embryonic lethality of ERK2 knockout mice 12.

**Figure 1:**
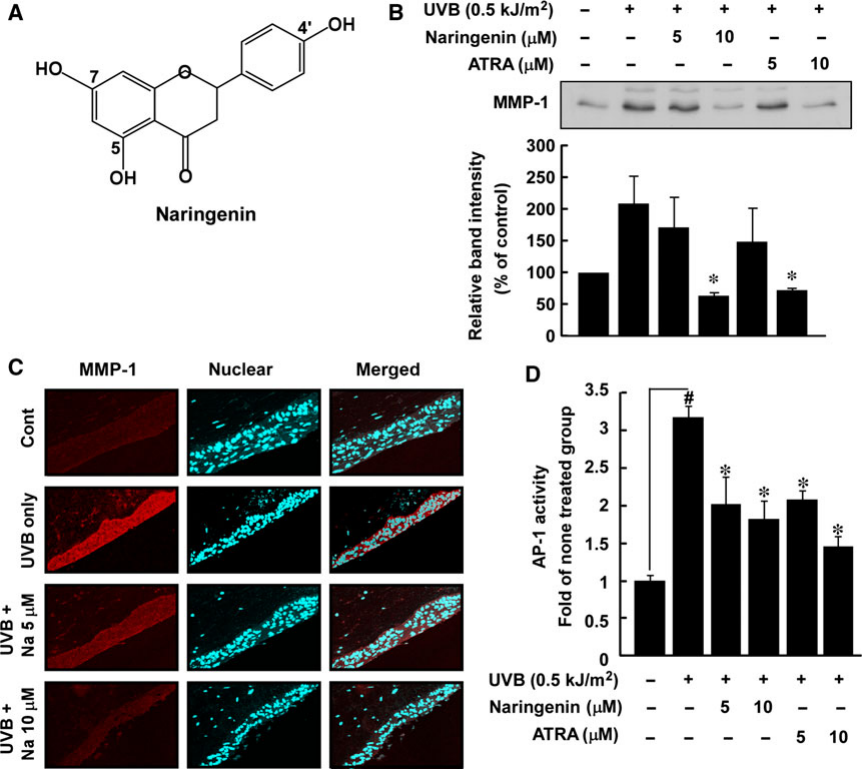
Effect of naringenin on UVB‐induced MMP‐1 expression and AP‐1 transactivation in HaCaT cells. (**A**) Chemical structure of naringenin (3,3′,4′,5,5′,7‐hexahydroxyflavone). (**B**) Naringenin and ATRA inhibit UVB‐induced MMP‐1 expression in HaCaT cells. The supernatant fraction was used for analysing MMP‐1 expression. Data are representative of three independent experiments that gave similar results and the asterisk (*) indicates a significant difference (*P* < 0.05) between treated and untreated cells. (**C**) Naringenin inhibits UVB‐induced MMP‐1 expression in 3‐D human skin equivalent cultures. The 3‐D human skin equivalent cultures were incubated for 10 days and treated with UVB alone or with naringenin and UVB. Samples were analysed by immunofluorescence to detect MMP‐1. Representative photos are shown. MMP‐1 expression is evident from the red staining. Nuclei in sections are counterstained with DAPI (blue). (**D**) Naringenin inhibits UVB‐induced AP‐1 luciferase activity. The AP‐1 luciferase assay is described in Materials and methods. Results are shown as mean values ± S.E.M. (*n* = 3). The symbol (#) indicates a significant difference (*P* < 0.05) between the control group and the UVB‐irradiated group. The asterisk (*) indicates a significant difference (*P* < 0.05) between groups treated with UVB irradiation and naringenin and the group treated with UVB alone.

Although ATRA is an effective and well‐known anti‐photoaging agent, several studies have indicated that it might have unfavourable side effects 14, 15. Flavonoids are benzo‐γ‐pyrone derivatives with various numbers of hydroxyl substitutions in their structures. Naringenin (3,3′,4′,5,5′,7‐hexahydroxyflavone) (Fig. 1A) is one of the major flavonoids found in several foods, including citrus. Naringenin was reported to decrease lung fibrosis and metastasis by down‐regulating transforming growth factor‐β1 and regulatory T cells in C57BL/6 mice 16. In a recent study, dietary supplementation with naringenin suppressed adiposity by increasing hepatic peroxisome proliferator‐activated receptor alpha protein expression and decreasing plasma triglycerides in male Long‐Evans hooded rats 17. In addition, the results from another study suggest that naringenin is a promising natural flavonoid for preventing skin ageng and carcinogenesis because of its protective effects against UVB‐induced apoptosis and cyclobutane pyrimidine dimers, which subsequently enhances long‐term survival of human HaCaT cells after UVB irradiation 18. These accumulated data provide evidence showing that naringenin might be an effective anti‐photoaging agent against UV irradiation, but the underlying mechanism and target(s) of naringenin remain unclear.

In this study, we show that naringenin suppresses UVB‐induced MMP‐1 expression and AP‐1 activity. These effects resulted from decreased FRA1 stability, which occurs when FRA1 phosphorylation at Ser265 is blocked through the direct repression of ERK2 activity.

## Materials and methods

### Reagents and antibodies

Naringenin (95%), and chemical reagents, including Tris, NaCl, SDS and cycloheximide (CHX) were purchased from Sigma‐Aldrich (St. Louis, MO, USA). Specific antibodies against MMP‐1 were purchased from Upstate Biotechnology (Lake Placid, NY, USA). Antibodies specific to detect Ser217/221‐phosphorylated MEK, total MEK, Thr359/Ser363‐phosphorylated p90^RSK^, total p90^RSK^, Tyr180/182‐phosphorylated p38, total p38, Ser63‐phosphorylated c‐Jun, total c‐Jun, total c‐Fos and Ser265‐phosphrylated FRA1 were from Cell Signaling Biotechnology (Beverly, MA, USA). Antibodies specific to Thr202/Tyr204‐phosphorylated ERK1/2, total ERKs, total FRA1, alpha tubulin and lamin B were obtained from Santa Cruz Biotechnology (Santa Cruz, CA, USA). The antibody against β‐actin was obtained from Sigma‐Aldrich. The recombinant active ERK2 protein was obtained from Upstate Biotechnology. CNBr‐Sepharose 4B, glutathione‐Sepharose 4B, [γ‐^32^P] ATP and the chemiluminescence detection kit were purchased from GE Healthcare (Piscataway, NJ, USA). The protein assay kit was obtained from Bio‐Rad Laboratories (Hercules, CA, USA).

### Cells culture, UVB exposure and transfection

The normal human epidermal HaCaT keratinocyte cell line and the normal BJ human fibroblast cell line were maintained in DMEM containing 10% foetal bovine serum (FBS; Atlanta Biologicals, Lawrenceville, GA, USA), 100 U/ml of penicillin, 100 mg/ml of streptomycin and Eagle's minimum essential medium (MEM) supplemented with 10% FBS and antibiotics, respectively, at 37°C in a 5% CO_2_ humidified incubator. UVB irradiation was applied using a bank of 4 Westinghouse F520 lamps (National Biological, Twinsburg, OH, USA) at 6 J/sec./m light in the UVB range. Approximately, 10% of the remaining radiation from the F520 lamp was in the UVA region (320 nm). The UVB exposure chamber was fitted with a Kodak Kodacel K6808 filter to eliminate all wavelengths below 290 nm. UVB radiation was measured using a UVX radiometer (UVX‐31). Transfection was performed with JetPEI (Polyplus‐Transfection, Inc., New York, NY, USA) following the manufacturer's protocol. The cells were cultured for 36–48 hrs and proteins extracted for further analysis. DNA used for CA‐MEK1 and DN‐ERK2 was prepared as described in a previous report 19.

### Western blot analysis

For MMP‐1 expression, cells were either treated or not treated with naringenin at the indicated concentrations (5 or 10 μM) for 1 hr, followed by UVB irradiation (0.5 kJ/m^2^). Aliquots of cell‐free supernatant fractions were collected 48 hrs after treatment. Then, 50 μg of protein were incubated with the MMP‐1 antibody at 4°C overnight. For other protein analysis, cells were either treated or not treated with naringenin at the indicated concentrations (5 or 10 μM) for 1 hr, followed by UVB irradiation (0.5 kJ/m^2^) and disruption with lysis buffer 11. Other processes were followed as reported previously 11.

### 3‐D organotypic culture

Construction and maintenance of organotypic skin cultures were performed essentially as described previously 20, 21 with certain modifications. Briefly, 3 ml of a collagen mixture containing 10× DME (0.3 ml), FBS (0.3 ml), 1 M NaOH (5 μl), collagen (1.7 ml), Fibroblast 650 (2 × 10^6^/ml) and DME (0.6 ml) were added onto a cell‐culture insert (# 161395; Nunc, Waltham, MA, USA) and allowed to gel by incubation for 1 hr at 37°C. After incubation, DMEM supplemented with 10% FBS 20 was added both above and below the collagen gel. After removing the media on top of the gel, keratinocytes (5 × 10^5^) were seeded onto the top surface. Thereafter, cells were treated or not treated with naringenin (5 or 10 μM) followed by UVB irradiation (0.5 kJ/m^2^) every day. After 10 days, the 3‐D organotypic culture was blocked with paraffin and sections (5 μm thick) of 10% neutral‐formalin‐solution‐fixed, paraffin‐embedded tissues were cut on silane‐coated glass slides for immunofluorescence analysis.

### Luciferase assay for AP‐1 transactivation

Confluent monolayers of HaCaT cells stably transfected with an AP‐1 luciferase plasmid 22 were harvested, and 8 × 10^3^ viable cells suspended in 100 μl of 10% FBS/DMEM were added to each well of a 96‐well plate. Plates were incubated at 37°C in 5% CO_2_. When cells reached 80–90% confluence, they were starved by culturing in 0.1% FBS–DMEM for another 24 hrs. The cells were treated for 1 hr with naringenin before exposure to UVB (0.5 kJ/m^2^), and then incubated for an additional 5 hrs. Cells were disrupted with 100 μl lysis buffer 11 and luciferase activity was measured using a luminometer (Luminoskan Ascent; Thermo Electron, Helsinki, Finland).

### UV irradiation of hairless mice

The animal experimental protocol (SNU‐120614‐3) was approved and animals were maintained under specific pathogen‐free conditions based on the guidelines established by the Animal Care and Use Committee of Korea Food Research Institute. Female SKH‐1 hairless mice (5 week old; mean bw, 25 g) were purchased from Central Laboratory Animal lnc. (Seoul, Korea). Animals were acclimated for 1 week prior to the study and had free access to food and water. The animals were housed in climate‐controlled quarters (24°C at 50% humidity) with a 12 hrs light/dark cycle. The UVB radiation source (Bio‐Link crosslinker; Vilber Lourmat, Torcy, France) emitted at wavelengths of 254, 312 and 365 nm, with peak emission at 312 nm. SKH‐1 mice were divided into six groups of five animals each: an untreated control group; a UVB‐treated group; two groups treated with UVB and different doses of naringenin; and one group treated with UVB and ATRA. We first measured the minimal erythema dose (MED) on mouse dorsal skin and defined 45 mJ/cm^2^ as one MED. The dorsal skin of hairless mice was exposed to UVB 3 times a week and the irradiation dose was increased weekly by 1 MED to 4 MED, and then maintained at 4 MED until 15 weeks.

### Determination of wrinkle area

After 15 weeks of treatment with naringenin, skin surface impressions were measured using silicon rubber (Silflo Dental Impression Materials, Potters Bar, UK) and analysed with Visioline VL650 (CK Electronics GmbH, Cologne, Germany).

### Evaluation of trans‐epidermal water loss and skin hydration

Trans‐epidermal Water loss (TEWL) and skin hydration of the mouse dorsal skin was measured with MoisturMap MM100, which was mounted on a Multi Probe Adapter MPA5 (CK Technology sprl, Rue de Maestricht, Belgium) and maintained at 20°C (±2°C) and 50% (±5%) humidity for 1 hr on the day before euthanasia for further experiments. TEWL and skin hydration were measured by vertically placing each probe on the dorsal skin of the mice, who were under anaesthesia. When the standard deviation of five or six continuously measured values was under 0.1, the average of the last five measured values was defined as the TEWL and skin hydration value for each mouse.

### Immunofluorescence

Five micro meter‐thick sections of paraffin‐embedded organotypic cultures and skin tissues were cut using a microtome and air‐dried at room temperature overnight. Paraffin sections were de‐paraffinized using xylene and then hydrated in descending grades of ethanol in distilled water, after which antigen retrieval was performed by heating samples at 95–100°C in 10 mM citrate buffer at pH 6 for 10 min. For immunofluorescence, sections from organotypic cultures were permeabilized and blocked at room temperature by incubating in PBS containing 0.02% Tween 20 and 1% bovine serum albumin (BSA) for 1 hr. Incubation with primary antibody diluted in PBS containing 3% BSA was performed at 4°C overnight. Secondary Texas red (Santa Cruz Biotechnology) antibodies were added at a 1:1000 dilution for 1 hr at room temperature. DAPI (1:10,000) was used as a nuclear stain. Sections were examined and photographed using either a Leica DM5000B fluorescence microscope (Leica, Wetzlar, Germany) or a Zeiss LSM 510s confocal microscope (Zeiss, Oberkochen, Germany). Images were analysed using Photoshop (Adobe, San Jose, CA, USA) software.

### RT‐PCR

Total RNA was isolated using the RNeasy^®^ Mini Kit (Qiagen, Valencia, CA, USA) according to the manufacturer's instructions. Reverse transcription of RNA was performed with the QuantiTect^®^ Reverse Transcription Kit (Qiagen). First‐strand cDNA was prepared from 1 μg of total RNA. The real‐time PCR reaction was performed in a volume of 25 μl containing 0.1 μg of cDNA, 1 μM of each primer (FRA1 sense 5′‐GAG CTG CAG TGG ATG GTA CA‐3′ and antisense 5′‐TGT ACC ATC CAC TGC AGC TC‐3′), and 2× Rotor‐Gene SYBR^®^ Green master mix. The thermal cycling was carried out in a Rotor‐Gene Q (Qiagen) with a program of 95°C for 5 min., followed by 40 cycles with denaturation at 95°C for 5 sec., annealing and elongation at 60°C for 10 sec. The gene expression levels were normalized to the expression level of the actin housekeeping gene (human actin sense 5′‐GAC GAT ATT GCC GCA CT‐3′ and antisense 5′‐GAT ACC ACG CTT GCT CTG AG‐3′). Relative gene expression changes, calculated using the 2^−∆∆CT^ method, are reported as number‐fold changes compared to those in the control samples.

### Preparation of naringenin–Sepharose 4B beads

A preparation of naringenin–Sepharose 4B freeze‐dried powder (0.3 g) was suspended in 1 mM HCl and the coupled solution [0.1 M NaHCO_3_ (pH 8.3) and 0.5 M NaCl] was mixed. The mixture was rotated end‐over‐end at 4°C overnight. The medium was transferred to 0.1 M Tris–HCl buffer (pH 8.0) and rotated end‐over‐end at 4°C overnight. The medium was washed 3 times with 0.1 M acetate buffer (pH 4.0) containing 0.5 M NaCl followed by a wash with 0.1 M Tris–HCl (pH 8.0) containing 0.5 M NaCl.

### *In vitro* MEK1 and ERK1/2 kinase assays

The *in vitro* MEK1 and ERK1/2 kinase assays were performed in accordance with the instructions provided by Upstate Biotechnology. In brief, every reaction solution contained 25 μl of assay reaction buffer 23 and a magnesium‐ATP cocktail buffer. For MEK1, 1 μg of inactive ERK2 peptide was included. A 4 μl aliquot was removed after incubating the reaction mixture at 30°C for 15 min., to which 20 μg of myelin basic protein substrate peptide for ERK2 and 10 μl of diluted [γ‐^32^P] ATP solution were added. For ERK1/2, 0.33 mg/ml of myelin basic protein substrate peptide was included. A 4 μl aliquot was removed after incubating the reaction mixture at 30°C for 15 min., to which 10 μl of diluted [γ‐^32^P] ATP solution were added. This mixture was incubated for 10 min. at 30°C, and then 25 μl aliquots were transferred onto p81 paper and washed three times with 0.75% phosphoric acid for 5 min. per wash and once with acetone for 2 min. The radioactive incorporation was determined using a scintillation counter. The effects of naringenin or ATRA were evaluated by separately incubating each compound with the reaction mixtures at 30°C for 15 min. Each experiment was performed three times.

### Co‐precipitation and ERK2 kinase assay

For immunoprecipitation, cells were either treated or not treated with naringenin at the indicated concentrations (5 or 10 μM) for 1 hr, followed by irradiation with UVB (0.5 kJ/m^2^) and disruption with lysis buffer 11. Lysate protein (500 μg) was cleared by A/G beads (20 μl) for 1 hr at 4°C in advance. The mixture was centrifuged at 16,128 *g* for 5 min. at 4°C, and the supernatant fraction was added to an ERK2 antibody (20 μl) and gently rocked overnight at 4°C. The pellets were suspended in kinase buffer 11 supplemented with 10 μl of diluted [γ‐^32^P] ATP solution and 10 μl of myelin basic protein. Reactions were conducted at 30°C for 30 min. and incorporated radioactivity was determined using a scintillation counter.

### Molecular docking

We used the Glide program for the docking study. The coordinates for the receptor co‐crystal structure (ERK2, PDB entry: 2OJG) were obtained from the protein databank. After removing all of the crystallographic water molecules, the protein structure was corrected by adding the missing atoms and then energy minimized with the OPLS‐AA force field. In the crystal structure, the co‐factor was bound to the ATP‐binding pocket. For docking of naringenin, a rectangular box surrounding the center of mass of the co‐factor in 2OJG.pdb (or the ATP‐binding pocket) was used to define the binding site. Glide has the capability to dock with different levels of precision scoring function. We used the extra precision (XP) scoring function, which provides the highest level of precision for ligand‐binding affinities. The final poses obtained from the Glide XP docking and their docking scores were used for structural analysis of the protein‐ligand complex.

### Statistical analysis

Data are expressed as mean values ± S.E.M. or S.D. as indicated. Statistical significance was determined using one‐way anova tests. Differences were considered significant at *P* < 0.05. All analyses were performed with Statistical Analysis Software (SAS, Inc., Cary, NC, USA).

## Results

### Naringenin inhibits UVB‐induced MMP‐1 expression and AP‐1 activity

Because abnormal induction of MMP‐1 plays an important role in UV‐induced photoaging through its degradation of collagen in dermal skin 1, we first examined the effect of naringenin on UVB‐induced MMP‐1 expression in human HaCaT cells. Naringenin (Fig. 1A) significantly inhibited UVB‐induced MMP‐1 expression, and its inhibitory effect on MMP‐1 expression was similar to ATRA, an FDA approved anti‐wrinkle agent (Fig. 1B). To further confirm the effect of naringenin on UVB‐induced MMP‐1 expression, we used 3‐D human skin equivalent cultures. Immunofluorescence data showed that treatment with naringenin suppressed UVB irradiation‐induced MMP‐1 expression (red colour) in human keratinocyte lesions (Fig. 1C). We further examined the activity of AP‐1, which is considered to be a major transcription factor for the *MMP‐1* gene 3, 22. Both naringenin and ATRA significantly reduced UVB‐induced transactivation of AP‐1 luciferase activity in HaCaT cells stably transfected with an AP‐1 luciferase plasmid (Fig. 1D), which agreed with previous findings 1.

### Naringenin suppresses UVB‐induced phosphorylation of p90^RSK^ and FRA1 expression

**Figure 2:**
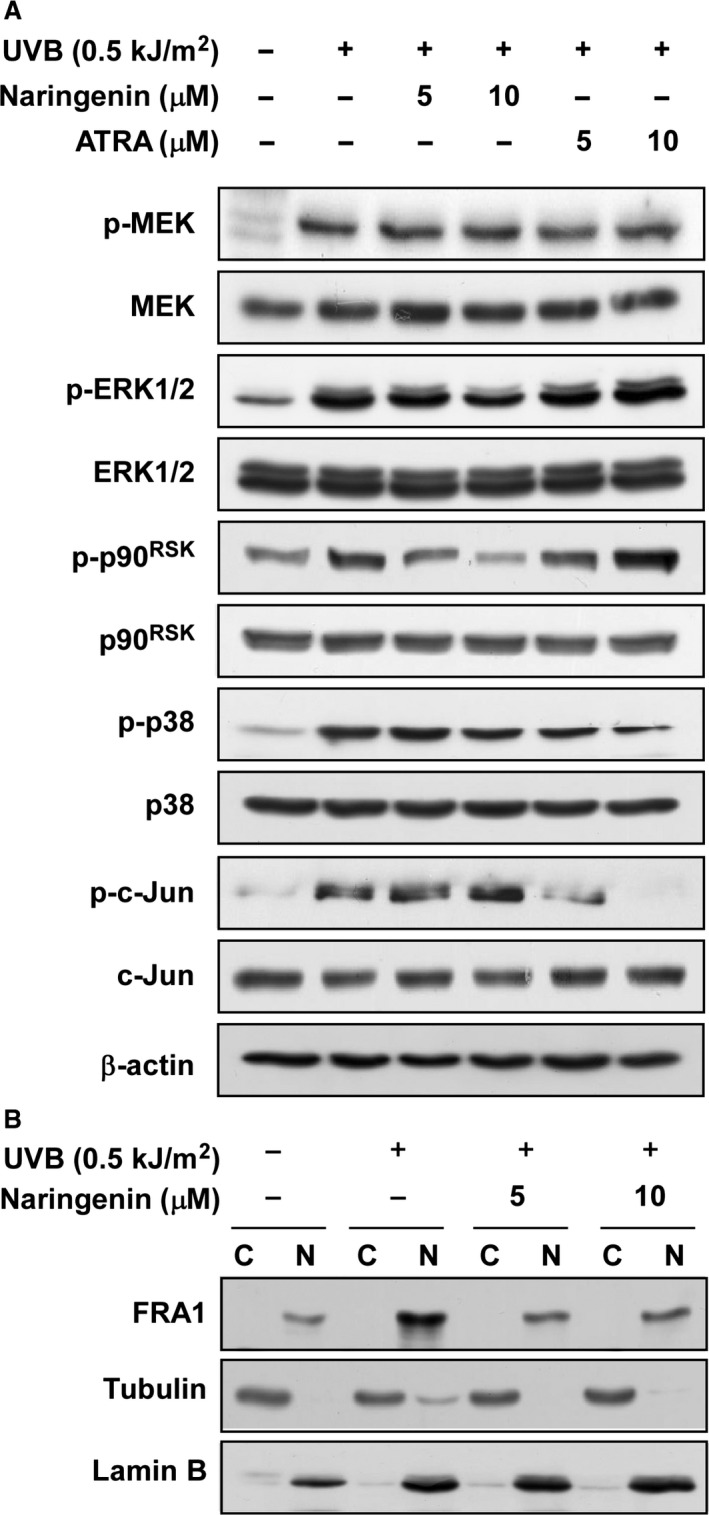
Effects of naringenin on UVB‐induced MAPKs signalling and FRA1 expression in HaCaT cells. (**A**) Naringenin inhibits UVB‐induced phosphorylation (*p*‐) of p90^RSK^ without affecting c‐Jun phosphorylation. The levels of phosphorylated and total MEK, ERK1/2, p90^RSK^ and p38 MAPK proteins and total c‐Fos were determined by Western blot analysis as described in Materials and methods. Data are representative of three independent experiments that gave similar results. (**B**) Naringenin inhibits UVB‐induced FRA1 expression in the nuclear fractions of HaCaT cells. Cytosolic and nuclear fractions were isolated as described in Materials and methods. FRA1 expression was detected in the cytosol and nucleus with specific antibodies. Tubulin and lamin B were used as a cytosolic and nucleic protein marker and loading controls respectively. Data are representative of three independent experiments that gave similar results.

Because ERKs signalling is a major regulator of FRA1 7, 8, 24, we investigated the possibility that naringenin could affect ERKs signalling. Results indicated that naringenin markedly inhibited UVB‐induced p90^RSK^ phosphorylation (Fig. 2A). We also found that naringenin attenuated UVB‐induced FRA1 expression (Fig. 2B), whereas the phosphorylation and expression of c‐Jun were not affected (Fig. 2A).

### Naringenin inhibits UVB‐induced FRA1 phosphorylation and decreases stability

**Figure 3:**
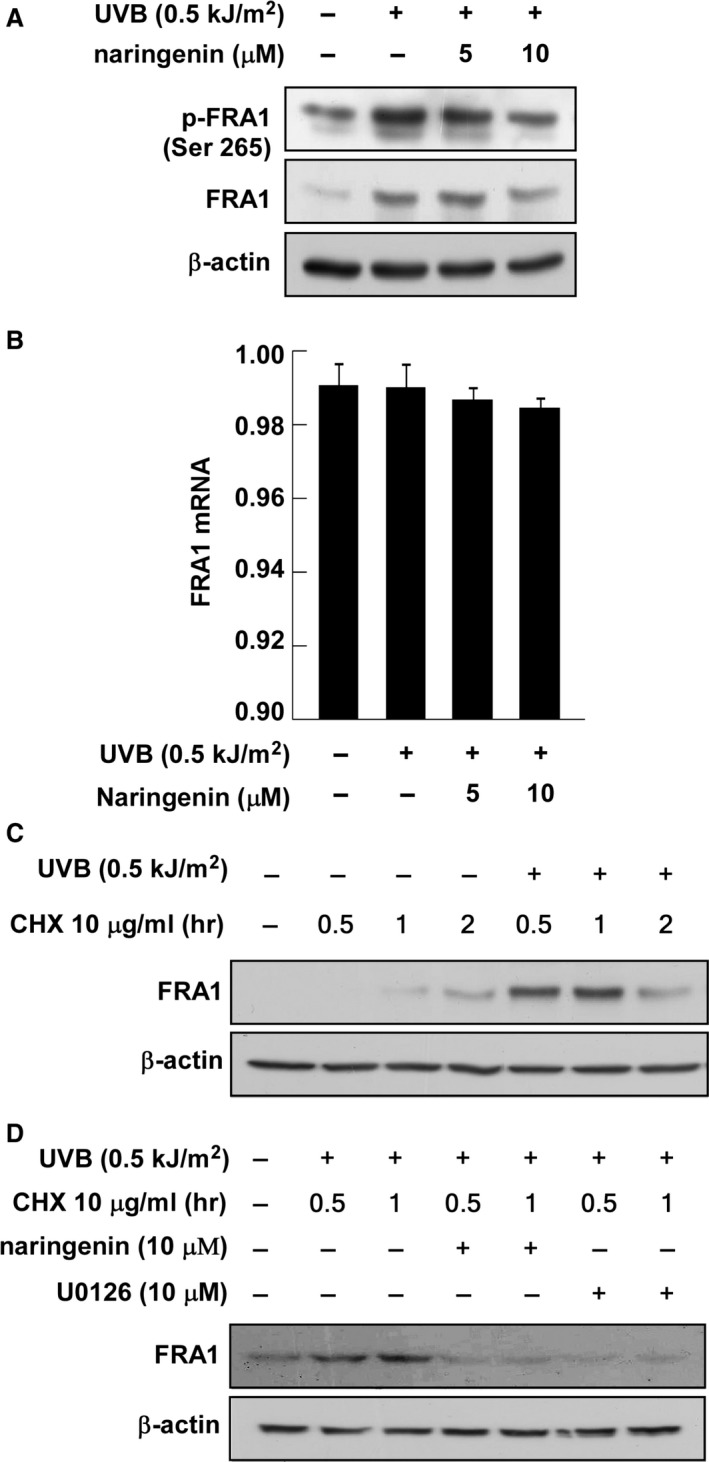
Effect of naringenin on UVB‐induced FRA1 mRNA and protein expression. (**A**) Naringenin inhibits UVB‐induced phosphorylation and expression of FRA1. Data are representative of three independent experiments that gave similar results. (**B**) Naringenin has no effect on UVB‐induced *FRA1* mRNA expression. Reverse transcription was conducted with random hexa‐primers, and FRA1 was amplified by PCR and visualized by ethidium bromide staining. Total RNA of β‐actin was resolved by electrophoresis and visualized as an internal control. Data are representative of three independent experiments that gave similar results. (**C**) UVB‐induced FRA1 protein expression is sustained for 1 hr after treatment with cycloheximide. Cells were exposed to UVB (0.5 kJ/m^2^) and then treated with cycloheximide (10 μg/ml) for 0.5, 1, or 2 hrs. FRA1 expression level was determined by Western blot analysis as described in Materials and methods. Data are representative of three independent experiments that gave similar results. (**D**) Naringenin or U0126 suppresses UVB‐induced FRA1 expression. Cells were pretreated with naringenin or U0126 for 1 hr and then were exposed to UVB (0.5 kJ/m^2^). Cells were then treated with cycloheximide (10 μg/ml) for 0.5 or 1 hr and harvested 12 hrs later. Data are representative of three independent experiments that gave similar results.

Western blot assay data showed that naringenin suppressed UVB‐induced FRA1 phosphorylation at Ser265 as well as FRA1 protein expression (Fig. 3A). We also investigated the possibility that naringenin regulates FRA1 at the transcriptional level. However, treatment with naringenin had no effect on *FRA1* mRNA levels (Fig. 3B). Based on this result and the fact that inhibition of FRA1 phosphorylation at Ser265 affects protein stability 7, we have been suggested that naringenin might influence FRA1 protein stability. To test our hypothesis, we examined FRA1 protein stability using CHX. Results indicated that FRA1 protein levels remained constant for 1 hr after co‐treatment with UVB and CHX (Fig. 3C). However, treatment with naringenin or U0126, a MEK inhibitor, decreased FRA1 protein levels compared to the untreated group (Fig. 3D).

### Naringenin directly inhibits ERK2 activity *in vitro* and *ex vivo*

**Figure 4:**
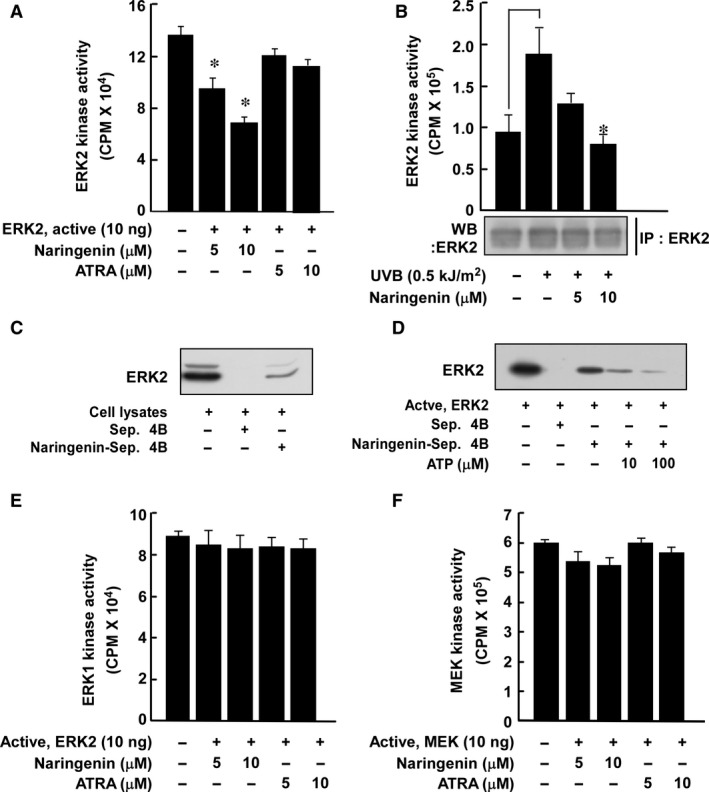
Effect of naringenin on MEK1 or ERK1/2 activity. (**A** and **B**) Naringenin inhibits ERK2 activity both (**A**) *in vitro* and (**B**) *ex vivo*. HaCaT cells were treated with naringenin as for Western blot assay conditions. Data are represented as mean values ± S.D. as determined from three independent experiments. For **A**, the asterisk (*) indicates a significant difference (*P* < 0.05) between the control group and the naringenin‐treated group. For **B**, the asterisk (*) indicates a significant difference (*P* < 0.05) between groups irradiated with UVB and treated with naringenin and the group exposed to UVB alone. (**C**) Naringenin directly binds to ERK2 in HaCaT cells. The *ex vivo* binding of naringenin and ERK2 was confirmed by immunoblotting using an antibody to detect ERK2. The first lane (input control): whole‐cell lysate from HaCaT cells; second lane (control): a lysate of HaCaT cells precipitated with Sepharose 4B beads as described in Materials and methods; third lane: whole‐cell lysate from HaCaT cells precipitated by naringenin–Sepharose 4B affinity beads as described in Materials and methods. (**D**) Naringenin directly binds to ERK2 in an ATP‐competitive manner. The mixtures including active ERK2, ATP (10 or 100 μM), and naringenin‐Sepharose 4B beads were incubated at 4°C overnight with shaking. After washing, the pulled‐down proteins were detected by Western blotting. Lane 1: ERK2 protein; lane 2: negative control, ERK2 cannot bind with Sepharose 4B beads; lane 3: positive control, ERK2 binds with naringenin–Sepharose 4B beads; lanes 4 and 5: increasing amounts of ATP decreased naringenin binding with ERK2. Each experiment was performed 3 times. (**E** and **F**) Naringenin has no effect on ERK1 or MEK1 kinase activity. Data are represented as mean values ± S.D. as determined from three independent experiments.

Because p90^RSK^ expression was markedly suppressed by naringenin, we have been suggested that naringenin might directly inhibit ERK1 or 2 activity. An ERK2 kinase assay and immunoprecipitation and kinase assay results each showed that naringenin inhibited UVB‐induced ERK2 activity *in vitro* and in HaCaT cells (Fig. 4A and B). A pull‐down assay with naringenin‐conjugated Sepharose 4B beads revealed that naringenin directly binds to ERK2 in an ATP‐competitive manner (Fig. 4C and D). However, naringenin had no effect on ERK1 or MEK1 activity (Fig. 4E and F).

### Inhibition of ERK signalling suppresses FRA1 phosphorylation and expression as well as MMP‐1 expression

**Figure 5:**
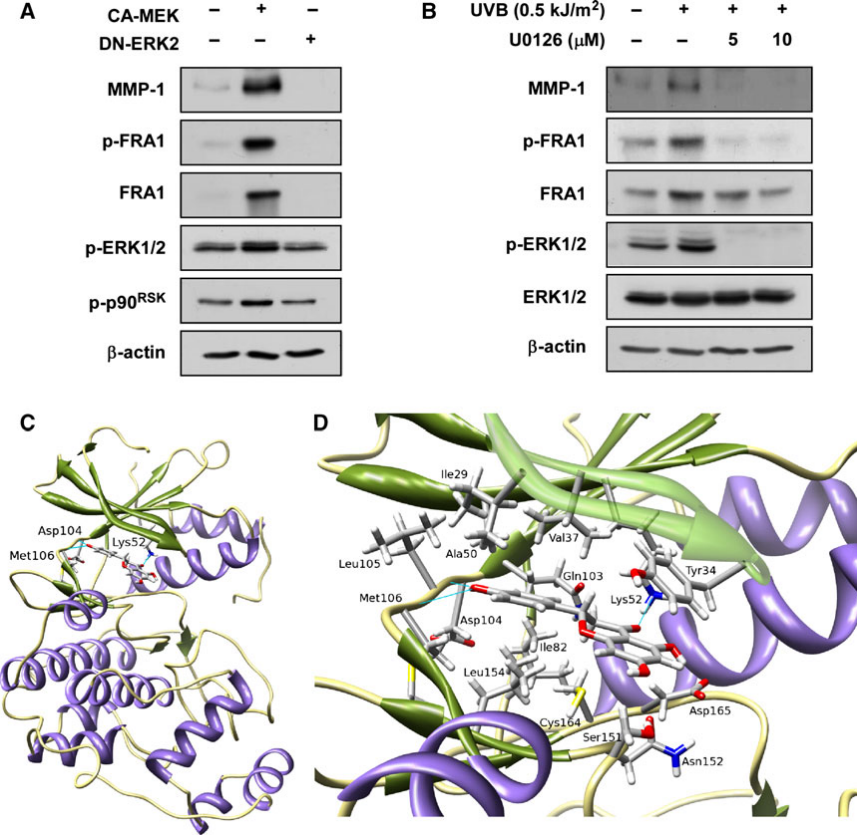
Effect of ERKs on MMP‐1 and FRA1 protein expression and docking of naringenin to ERK2. (**A**) CA‐MEK increases MMP‐1 expression as well as FRA1 phosphorylation and expression, whereas dominant‐negative (DN) ERK2 inhibits MMP‐1 expression as well as FRA1 phosphorylation and expression. Cells were harvested 24 hrs after transfection with the indicated plasmid and MMP‐1 expression and FRA1 phosphorylation and expression were determined by Western blotting. Data are representative of 3 independent experiments that gave similar results. (**B**) U0126 inhibits UVB‐induced MMP‐1 expression as well as FRA1 phosphorylation and expression in HaCaT cells. Cells were pretreated with U0126 at the indicated concentrations (0, 5 or 10 μM) for 1 hr, then stimulated with UVB (0.5 kJ/m^2^) and harvested 30 min. or 48 hrs later. Data are representative of three independent experiments that gave similar results. (**C**) Naringenin docks into the ATP pocket of ERK2. Hydrogen bonds between naringenin and amino acids are shown in a stick model. All of the hydrogen bond interactions between naringenin and the amino acid residues Asp104, Met106, and Lys52 are shown as blue lines. (**D**) Enlarged view of ERK2‐narigenin interactions. Amino acids (Val, Ala, Ile, and Leu) that form the hydrophobic pocket around naringenin and the amino acids that form favourable electrostatic interactions (Asp165 and Ser151) are shown in stick model.

To confirm the influence of ERK2 and FRA1 signalling on MMP‐1 expression, we used a constitutively active MEK (CA‐MEK) plasmid, a dominant‐negative ERK2 (DN‐ERK2) plasmid, or the pharmacological MEK inhibitor, U0126. The results showed that CA‐MEK induced FRA1 phosphorylation and expression as well as increased MMP‐1 expression, whereas DN‐ERK2 had no effect on FRA1 or MMP‐1 (Fig. 5A). In addition, inhibition of ERKs signalling using the U0126 compound also suppressed UVB‐induced FRA1 phosphorylation and expression and also decreased MMP‐1 expression (Fig. 5B). Computer modelling results indicated that naringenin docks into the ATP pocket of ERK2 (Fig. 5C and D).

### Naringenin suppresses UVB‐induced wrinkle formation, TEWL and MMP‐13 expression *in vivo*

**Figure 6:**
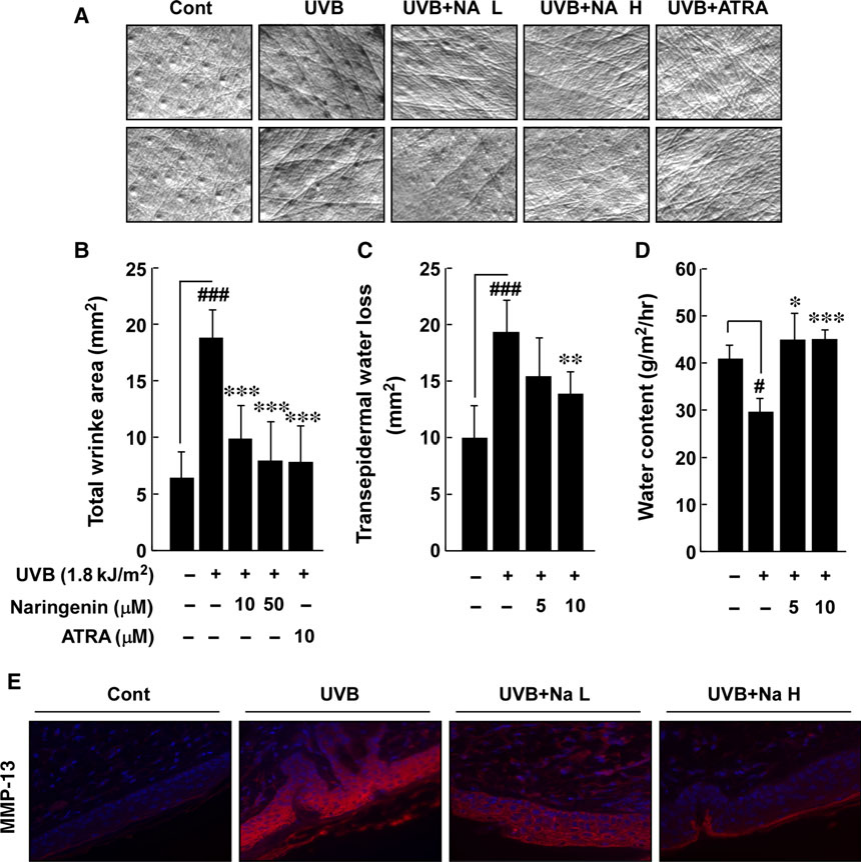
Effect of naringenin on UVB‐induced wrinkle formation, trans‐epidermal water loss (TEWL), water content, and MMP‐13 expression in the SKH‐1 hairless mouse model. (**A**) External appearance of wrinkling. (**B**) Naringenin significantly inhibits UVB‐induced wrinkle formation in the SKH‐1 hairless mouse model. Five mice received a topical treatment of 200 μl of acetone alone in the unexposed control group. In the UVB‐treated group, the mice were topically treated with 200 μl of acetone before UVB exposure for 15 weeks. The mice in the third and fourth groups received topical application of naringenin and the fifth group received topical application of ATRA for 15 weeks 1 hr before UVB irradiation. The frequency of irradiation was set at three times per week. Respective doses of naringenin (10 or 50 μM in 200 μl acetone/mouse) or ATRA (10 μM in 200 μl acetone/mouse) and UVB (1.8 kJ/cm^2^) were topically applied to the dorsal area. (**C**) TEWL and (**D**) skin hydration were measured by Corneometer and Tewameter 3 days before the animals were killed. (**E**) Naringenin inhibits UVB‐induced MMP‐13 expression in SKH‐1 hairless mice. Samples were analysed by immunofluorescence to detect MMP‐13. Representative photos are shown. MMP‐13 expression is evident from the red staining. Nuclei in sections were counterstained with DAPI (blue). Results (**B**–**D**) are shown as mean values ± S.E.M. (*n* = 5). The symbols (#) and (###) indicate a significant difference (*P* < 0.05) and (*P* < 0.001) between the control group and the UVB‐irradiated group. The asterisks (*), (**) and (***) indicate a significant difference (*P* < 0.05), (*P* < 0.01), and (*P* < 0.001) between groups treated with UVB irradiation and naringenin and the group treated with UVB alone.

The SKH‐1 hairless mouse model has been recommended to investigate the anti‐photoaging effects of natural phytochemicals 25. We adopted this model to determine whether naringenin could prevent photoaging caused by long‐term exposure to UVB. Chronic irradiation of mouse skin to UVB induced wrinkle formation and treatment with naringenin significantly suppressed UVB‐induced wrinkle formation (Fig. 6A and B). Notably, the inhibitory effect on UVB‐induced wrinkle formation was similar to that of ATRA (Fig. 6A and B). In addition, naringenin suppressed UVB‐induced epidermal water loss (Fig. 6C) and increased water content (Fig. 6D) compared to the group treated with only UVB. Immunofluorescence analysis for MMP‐13 clearly showed that naringenin strongly suppressed UVB‐induced MMP‐13 expression in mouse skin (Fig. 6E).

## Discussion

Accumulating evidence from *in vivo* human skin studies has shown that ATRA suppresses UV‐induced premature skin ageing, referred to as photoaging, by inhibiting MMP‐1 expression 1, 26. All‐trans retinoic acid, however, can induce retinoic acid syndrome 14 and epidermal hyperplasia, which can lead to excessive scaling 15. Therefore, effective and safe anti‐photoaging agents are still needed. Most natural phytochemicals are considered to be safe for therapeutic applications because of their long history of human consumption 27. In animal models, we also have shown that certain phytochemicals have anti‐skin cancer and anti‐wrinkling properties with no side effects 11, 28, 29. In addition, phytochemicals can act as small molecule inhibitors of cancer 30, 31. Therefore, targeting wrinkle‐inducing signal pathways using natural phytochemicals is a promising strategy for the prevention or attenuation of photoaging.

Matrix metalloproteinase‐1 acts as a major mediator of UV‐induced photoaging by degrading the collagen in human skin 1, 3. Therefore, if specific natural phytochemicals suppress UV‐induced MMP‐1 expression, they could be promising anti‐photoaging agents. We found that naringenin effectively inhibits UVB‐induced MMP‐1 expression in human skin HaCaT keratinocytes and the effect was similar to the effect of ATRA. A study featuring 3‐D human skin equivalent cultures also showed that naringenin suppressed UVB‐induced MMP‐1 expression. Furthermore, naringenin or ATRA significantly inhibited UVB‐induced AP‐1 activity. These results suggested that inhibition of UVB‐induced MMP‐1 expression might be because of suppression of AP‐1 transactivation.

According to previous study results, phosphorylation and expression of the c‐Jun and c‐Fos family proteins play a major role in AP‐1 activity and MMP‐1 expression 26, 32. Our observations indicate, however, that naringenin only suppresses UVB‐induced FRA1 expression without any measurable effect on c‐Jun or p38 phosphorylation. Knockdown of FRA1 reduces Raf1‐induced AP‐1 activity and MMP‐1 expression, implying that FRA1 plays a major role 8. Therefore, these results indicate that naringenin inhibits UVB‐induced AP‐1 activity and MMP‐1 expression by suppressing FRA1 expression. However, because the mechanism by which UVB‐induced FRA1 is influenced by naringenin in human keratinocytes remains unknown, we examined the transcriptional regulation of FRA1. Our RT‐PCR data showed that naringenin did not alter *FRA1* mRNA levels. A previously published paper suggested that protein degradation is a major regulatory mechanism of FRA1, and phosphorylation of FRA1 at Ser265 is critical for protein stability 7. Our Western blot assay results show that naringenin suppressed the expression and UVB‐induced phosphorylation of FRA1 at Ser265 (Fig. 3A). Moreover, FRA1 protein stability induced by co‐treatment with UVB and CHX was decreased by naringenin or U0126. Others also reported that treatment with an RSK inhibitor, fmk, reduced FRA1 protein levels by 60%, whereas its transcription was unaffected 8, 10. This evidence and our results indicate that naringenin mediates FRA1 stability by inhibiting phosphorylation at Ser265, subsequently affecting AP‐1 and MMP‐1 expression.

Our Western blot analysis indicated that naringenin inhibited UVB‐induced p90^RSK^ phosphorylation without affecting ERK1/2 phosphorylation. These results suggested that naringenin might directly target ERK1/2. Indeed, naringenin specifically inhibited ERK2 activity without affecting ERK1 (Fig. 4B). Moreover, the results of our pull‐down assay showed direct binding between ERK2 and naringenin‐Sepharose 4B beads. To confirm this result, we performed experiments using CA‐MEK and DN‐ERK2. CA‐MEK induced FRA1 phosphorylation and expression as well as increased MMP‐1 expression, whereas DN‐ERK2 had no effect. UVB‐induced FRA1 phosphorylation and expression as well as MMP‐1 expression were strongly inhibited by a specific MEK inhibitor, U0126. In our ERK2 kinase and pull‐down assays, naringenin substantially inhibited UVB‐induced ERK2 activity by binding to ERK2. Therefore, naringenin could be a major regulator of MMP‐1 expression acting by mediating FRA1 and AP‐1. Importantly, naringenin suppressed chronic UVB‐irradiation‐induced wrinkle formation in mouse skin. Although in our previous studies, we observed that chronic UVB (180 mJ/cm^2^) induced skin tumour development 11, we did not observe the appearance of skin tumours in our photoaging model. This might have been due to the weekly increase in the UVB dose from 45 mJ/cm^2^ (1 MED) to (4 MED). The 4 MED dose was only continued for a short period of time and thus, was not sufficient to induce skin tumours.

The docking conformation of naringenin is shown (Fig. 5C) in its most favoured binding mode in the ATP site, which is located at the interface between the N‐ and C‐terminal lobes of ERK2. The 4′‐hydroxyl group of naringenin forms two hydrogen bonds with the hinge region of ERK2 that involves the backbone ‐CO of Asp104 and the backbone ‐NH of Met106. Naringenin is further stabilized by a hydrogen bond formed between its chroman‐4‐one and the ‐NH_3_ of Lys52 in the N‐lobe of ERK2 (see close‐up view Fig. 5D). The upper and lower areas of naringenin were stabilized primarily by hydrophobic interactions involving the hydrocarbon side chains of Val, Ala, Leu, and Ile. The gatekeeper residue Gln103, adjacent to naringenin, made favourable hydrophobic interactions with the aromatic ring of naringenin. Thus, the modelling study showed that naringenin could bind to the ATP‐binding pocket of ERK2, which is consistent with the experimental observation that naringenin competitively blocks ATP‐binding and ERK2 activity.

In summary, we have shown that naringenin strongly inhibited UVB‐induced MMP‐1 expression in a 3‐D human skin equivalent culture and MMP‐13 expression and wrinkle formation in SKH‐1 hairless mice. This inhibitory effect occurred mainly through the blocking of ERK2 kinase activity, suggesting that ERK2 is a critical target for naringenin in mediating FRA1 stability and AP‐1 activity. Therefore, ERK2‐mediated FRA1 stability appears to be very important for UVB‐induced MMP‐1 expression and wrinkle formation. Therapeutic inhibition of ERK2 by naringenin might have preventive effects against photoaging.

## Conflicts of interest

The authors have declared no conflict of interest.

## Author contribution

Sung Keun Jung created figures and wrote the initial manuscript text; Su Jeong Ha, Chang Hwa Jung, Yun Tai Ki, Hoo‐Keun Lee, Myoung Ok Kim, MeeHyun Lee helped conduct the *in vivo* studies and Western blot assay figures; Madhusoodanan Mottamal created the docking model between naringenin and ERK2. All authors discussed the results, supported experiments, and commented on the manuscript. Ann M. Bode, Ki Won Lee and Zigang Dong supervised and ensured implementation of the project.

## References

[1] Fisher GJ , Datta SC , Talwar HS , *et al* Molecular basis of sun‐induced premature skin ageing and retinoid antagonism. Nature. 1996; 379: 335–9. 8552187 10.1038/379335a0

[2] Silvers AL , Bowden GT . UVA irradiation‐induced activation of activator protein‐1 is correlated with induced expression of AP‐1 family members in the human keratinocyte cell line HaCaT. Photochem Photobiol. 2002; 75: 302–10. 11950097 10.1562/0031-8655(2002)075<0302:uiiaoa>2.0.co;2

[3] Brennan M , Bhatti H , Nerusu KC , *et al* Matrix metalloproteinase‐1 is the major collagenolytic enzyme responsible for collagen damage in UV‐irradiated human skin. Photochem Photobiol. 2003; 78: 43–8. 12929747 10.1562/0031-8655(2003)078<0043:mmitmc>2.0.co;2

[4] Tanaka K , Asamitsu K , Uranishi H , *et al* Protecting skin photoaging by NF‐kappaB inhibitor. Curr Drug Metab. 2010; 11: 431–5. 20540695 10.2174/138920010791526051

[5] Oplander C , Hidding S , Werners FB , *et al* Effects of blue light irradiation on human dermal fibroblasts. J Photochem Photobiol B Biol. 2011; 103: 118–25. 10.1016/j.jphotobiol.2011.02.018 21421326

[6] Chung L , Dinakarpandian D , Yoshida N , *et al* Collagenase unwinds triple‐helical collagen prior to peptide bond hydrolysis. EMBO J. 2004; 23: 3020–30. 15257288 10.1038/sj.emboj.7600318 PMC514933

[7] Basbous J , Chalbos D , Hipskind R , *et al* Ubiquitin‐independent proteasomal degradation of Fra‐1 is antagonized by Erk1/2 pathway‐mediated phosphorylation of a unique C‐terminal destabilizer. Mol Cell Biol. 2007; 27: 3936–50. 17371847 10.1128/MCB.01776-06 PMC1900028

[8] Doehn U , Hauge C , Frank SR , *et al* RSK is a principal effector of the RAS‐ERK pathway for eliciting a coordinate promotile/invasive gene program and phenotype in epithelial cells. Mol Cell. 2009; 35: 511–22. 19716794 10.1016/j.molcel.2009.08.002 PMC3784321

[9] Shin S , Dimitri CA , Yoon SO , *et al* ERK2 but not ERK1 induces epithelial‐to‐mesenchymal transformation *via* DEF motif‐dependent signaling events. Mol Cell. 2010; 38: 114–27. 20385094 10.1016/j.molcel.2010.02.020 PMC2854677

[10] Eferl R , Wagner EF . AP‐1: a double‐edged sword in tumorigenesis. Nat Rev Cancer. 2003; 3: 859–68. 14668816 10.1038/nrc1209

[11] Jung SK , Lee KW , Byun S , *et al* Myricetin suppresses UVB‐induced skin cancer by targeting Fyn. Cancer Res. 2008; 68: 6021–9. 18632659 10.1158/0008-5472.CAN-08-0899

[12] Satoh Y , Endo S , Ikeda T , *et al* Extracellular signal‐regulated kinase 2 (ERK2) knockdown mice show deficits in long‐term memory; ERK2 has a specific function in learning and memory. J Neurosci. 2007; 27: 10765–76. 17913910 10.1523/JNEUROSCI.0117-07.2007 PMC6672813

[13] Pages G , Guerin S , Grall D , *et al* Defective thymocyte maturation in p44 MAP kinase (Erk 1) knockout mice. Science. 1999; 286: 1374–7. 10558995 10.1126/science.286.5443.1374

[14] Altucci L , Gronemeyer H . The promise of retinoids to fight against cancer. Nat Rev Cancer. 2001; 1: 181–93. 11902573 10.1038/35106036

[15] Rittie L , Varani J , Kang S , *et al* Retinoid‐induced epidermal hyperplasia is mediated by epidermal growth factor receptor activation *via* specific induction of its ligands heparin‐binding EGF and amphiregulin in human skin *in vivo* . J Invest Dermatol. 2006; 126: 732–9. 16470170 10.1038/sj.jid.5700202

[16] Du G , Jin L , Han X , *et al* Naringenin: a potential immunomodulator for inhibiting lung fibrosis and metastasis. Cancer Res. 2009; 69: 3205–12. 19318568 10.1158/0008-5472.CAN-08-3393

[17] Cho KW , Kim YO , Andrade JE , *et al* Dietary naringenin increases hepatic peroxisome proliferators‐activated receptor alpha protein expression and decreases plasma triglyceride and adiposity in rats. Eur J Nutr. 2011; 50: 81–8. 20567977 10.1007/s00394-010-0117-8

[18] El‐Mahdy MA , Zhu Q , Wang QE , *et al* Naringenin protects HaCaT human keratinocytes against UVB‐induced apoptosis and enhances the removal of cyclobutane pyrimidine dimers from the genome. Photochem Photobiol. 2008; 84: 307–16. 18086244 10.1111/j.1751-1097.2007.00255.x PMC2756997

[19] Zhu F , Zykova TA , Kang BS , *et al* Bidirectional signals transduced by TOPK‐ERK interaction increase tumorigenesis of HCT116 colorectal cancer cells. Gastroenterology. 2007; 133: 219–31. 17631144 10.1053/j.gastro.2007.04.048

[20] Okugawa Y , Hirai Y . Overexpression of extracellular epimorphin leads to impaired epidermal differentiation in HaCaT keratinocytes. J Invest Dermatol. 2008; 128: 1884–93. 18273050 10.1038/jid.2008.22

[21] Martins VL , Vyas JJ , Chen M , *et al* Increased invasive behaviour in cutaneous squamous cell carcinoma with loss of basement‐membrane type VII collagen. J Cell Sci. 2009; 122: 1788–99. 19435799 10.1242/jcs.042895 PMC2684833

[22] Chen W , Borchers AH , Dong Z , *et al* UVB irradiation‐induced activator protein‐1 activation correlates with increased c‐fos gene expression in a human keratinocyte cell line. J Biol Chem. 1998; 273: 32176–81. 9822695 10.1074/jbc.273.48.32176

[23] Lee KW , Kang NJ , Rogozin EA , *et al* Myricetin is a novel natural inhibitor of neoplastic cell transformation and MEK1. Carcinogenesis. 2007; 28: 1918–27. 17693661 10.1093/carcin/bgm110

[24] Gullett NP , Ruhul Amin AR , Bayraktar S , *et al* Cancer prevention with natural compounds. Semin Oncol. 2010; 37: 258–81. 20709209 10.1053/j.seminoncol.2010.06.014

[25] Park JY , Jang YH , Kim YS , *et al* Ultrastructural changes in photorejuvenation induced by photodynamic therapy in a photoaged mouse model. Eur J Dermatol. 2013; 23: 471–7. 23797711 10.1684/ejd.2013.2050

[26] Fisher GJ , Talwar HS , Lin J , *et al* Retinoic acid inhibits induction of c‐Jun protein by ultraviolet radiation that occurs subsequent to activation of mitogen‐activated protein kinase pathways in human skin *in vivo* . J Clin Invest. 1998; 101: 1432–40. 9502786 10.1172/JCI2153 PMC508699

[27] Surh YJ . Cancer chemoprevention with dietary phytochemicals. Nat Rev Cancer. 2003; 3: 768–80. 14570043 10.1038/nrc1189

[28] Jung SK , Lee KW , Byun S , *et al* Myricetin inhibits UVB‐induced angiogenesis by regulating PI‐3 kinase *in vivo* . Carcinogenesis. 2010; 31: 911–7. 20008033 10.1093/carcin/bgp221 PMC2864405

[29] Jung SK , Lee KW , Kim HY , *et al* Myricetin suppresses UVB‐induced wrinkle formation and MMP‐9 expression by inhibiting Raf. Biochem Pharmacol. 2010; 79: 1455–61. 20093107 10.1016/j.bcp.2010.01.004 PMC2864126

[30] Lee KW , Bode AM , Dong Z . Molecular targets of phytochemicals for cancer prevention. Nat Rev Cancer. 2011; 11: 211–8. 21326325 10.1038/nrc3017

[31] Kang NJ , Shin SH , Lee HJ , *et al* Polyphenols as small molecular inhibitors of signaling cascades in carcinogenesis. Pharmacol Ther. 2011; 130: 310–24. 21356239 10.1016/j.pharmthera.2011.02.004

[32] Milde‐Langosch K . The Fos family of transcription factors and their role in tumourigenesis. Eur J Cancer. 2005; 41: 2449–61. 16199154 10.1016/j.ejca.2005.08.008

